# Using transcriptome profiling to characterize QTL regions on chicken chromosome 5

**DOI:** 10.1186/1471-2164-10-575

**Published:** 2009-12-02

**Authors:** Guillaume Le Mignon, Colette Désert, Frédérique Pitel, Sophie Leroux, Olivier Demeure, Gregory Guernec, Behnam Abasht, Madeleine Douaire, Pascale Le Roy, Sandrine Lagarrigue

**Affiliations:** 1 INRA, UMR598, Génétique Animale, IFR140 GFAS, F-35000 Rennes, France; 2 Agrocampus Ouest, UMR598, Génétique Animale, IFR140 GFAS, F-35000 Rennes, France; 3 ITAVI, F-75008, Paris, France; 4 INRA, UR444, Laboratoire de Génétique Cellulaire, F-31326 Auzeville, France; 5 INRA, UR1012 SCRIBE, IFR140, GenOuest, 35000 Rennes, France

## Abstract

**Background:**

Although many QTL for various traits have been mapped in livestock, location confidence intervals remain wide that makes difficult the identification of causative mutations. The aim of this study was to test the contribution of microarray data to QTL detection in livestock species. Three different but complementary approaches are proposed to improve characterization of a chicken QTL region for abdominal fatness (AF) previously detected on chromosome 5 (GGA5).

**Results:**

Hepatic transcriptome profiles for 45 offspring of a sire known to be heterozygous for the distal GGA5 AF QTL were obtained using a 20 K chicken oligochip. mRNA levels of 660 genes were correlated with the AF trait. The first approach was to dissect the AF phenotype by identifying animal subgroups according to their 660 transcript profiles. Linkage analysis using some of these subgroups revealed another QTL in the middle of GGA5 and increased the significance of the distal GGA5 AF QTL, thereby refining its localization. The second approach targeted the genes correlated with the AF trait and regulated by the GGA5 AF QTL region. Five of the 660 genes were considered as being controlled either by the AF QTL mutation itself or by a mutation close to it; one having a function related to lipid metabolism (HMGCS1). In addition, a QTL analysis with a multiple trait model combining this 5 gene-set and AF allowed us to refine the QTL region. The third approach was to use these 5 transcriptome profiles to predict the paternal Q versus q AF QTL mutation for each recombinant offspring and then refine the localization of the QTL from 31 cM (100 genes) at a most probable location confidence interval of 7 cM (12 genes) after determining the recombination breakpoints, an interval consistent with the reductions obtained by the two other approaches.

**Conclusion:**

The results showed the feasibility and efficacy of the three strategies used, the first revealing a QTL undetected using the whole population, the second providing functional information about a QTL region through genes related to the trait and controlled by this region (HMGCS1), the third could drastically refine a QTL region.

## Background

In spite of success in QTL research for complex traits in livestock species in the last twenty years, location confidence intervals of many QTL are wide, possibly harboring hundreds of genes. This is the major obstacle to finding causative mutations underlying any QTL identified. In addition, fine mapping techniques and positional cloning to reduce the location confidence interval of the initial QTL are time-consuming, especially for livestock species compared to plant and animal models. This is mainly due to a lack of inbred lines, long generation intervals, the cost of maintaining each animal and also the difficulty of producing transgenic or "knock-out" individuals to confirm the causative nature of the mutation of the trait of interest. Few mutations underlying QTL have therefore been identified in livestock (*e.g*., the *DGAT1 *gene in dairy cattle [1], *IGF2 *gene in swine [2,3], *GDF8 *gene in sheep [4]*etc*, for review see Ron & Weller [5] and Georges [6]). Several groups have proposed combining QTL detection programs and high throughput transcriptome data to elucidate biological pathways associated with complex traits and their underlying genetic determinants. [7-14]. This new integrative approach, known as "Genetical Genomics (GG)" or "Integrative Genomics", treats the expression level of each gene present on a microarray as a quantitative trait and use genetic markers to identify genomic regions that regulate gene expression phenotypes. Such regions are named eQTL (expression Quantitative Trait Loci). Independently of the context of QTL identification for a complex trait, the eQTL identification approach was first applied in 2002 by Brem *et al*. [15] in order to understand the genetic architecture of natural variations in gene expression in yeast. This approach was soon extended to eukaryotes [10,11,15-20]. An eQTL region close to the physical location of a gene controlled by this region is referred to as a cis-eQTL [10,15]. In such a case, a mutation in the gene itself might be responsible for variability in its own expression at the mRNA level. When an eQTL region for a given gene maps to a location on the genome other than the localization of this gene, it is referred to as a trans e-QTL. Very little is known of the molecular nature of cis-acting and (even more so) trans-acting eQTL regions.

In the context of QTL identification for a complex trait, GG studies have mainly been undertaken on plant or animal models such as flies [12], mice [10], rats [11], eucalyptus [13], Arabidopsis [14]. GG is not yet usually used because it requires skills in both genetic and genomic fields and the cost of microarray is high, which can be a real limitation for GG in which several animals have to be analyzed. The present study aims at testing the microarray contribution to a QTL research program in livestock species in which the population structure and marker density are less favorable compared to animal models to QTL localization (no consanguineous lines, low marker density...). Our question was therefore whether GG could be transposed in livestock species to reduce a QTL region of interest and to provide new functional information about the causative mutation. To answer this question, this study focused on chicken species, with abdominal fatness as the complex trait. Although various QTL for the fatness trait have been reported in this species [21], these QTL regions remain wide and no causative mutation has been clearly detected. We chose to apply three complementary strategies integrating transcriptome data in order to improve characterization of a QTL for abdominal fatness (AF) previously detected on the chicken chromosome GGA5 (p < 0.01) with an effect of 1 phenotypic standard deviation [22]. Because of the cost of microarray, the experimental design in the present study included 45 birds. Preliminary linkage analysis on this design revealed the expected AF QTL on the distal GGA5 (p < 0.05) showing that, despite the fairly small size of the experimental design, it is possible to detect QTL with an effect of ~1 phenotypic standard deviation, thus justifying continuing the study. One of the strategies used was based on dissection of the complex trait using the elementary gene expression profiles, as first performed in 2003 by Schadt *et al*. [10]. To the best to our knowledge, no study using this approach has been published since this first report. The second strategy commonly used by authors working in this context was based on the identification of genes with eQTL co-localizing with the QTL responsible for the complex trait of interest. The function of such genes can provide new functional information about the candidate positional and functional gene sought in the QTL region as causative to the trait of interest. Only one study conducted in livestock species has been reported using this approach [23]. In the present study, we used this strategy in this way in order to characterize the QTL functionally; we also used this strategy in another way using a multiple trait model for QTL analysis in order to refine this QTL region. A third approach was to use hepatic transcriptome profiles to predict for each recombinant offspring the Q versus q GGA5 AF QTL allele (at the mutation looked for) inherited from its sire and then to refine the localization of the GGA5 AF QTL after determining the recombination breakpoints.

## Results

### Animal design and microarray setup

Previous studies using a three-generation design performed by inter-crossing two experimental chicken lines divergently selected on abdominal fatness have revealed 6 QTL for abdominal fatness (AF) on GGA1, 3, 5 and 7 in male meat-type chickens, [22,24]. Different recombinant backcross 1 (BC1) and BC2 males were then produced to refine AF QTL on chromosome 5 by crossing F1 sires heterozygous for these QTL with lean line dams [22,24]. The present study focused on a BC1 chicken sire (and its 71 BC2 male offspring) known to be heterozygous for the AF QTL on the chicken distal GGA5 chromosome, the other QTL on GGA1, 3 and 7 were not detected in this family. Our aim was to integrate hepatic transcriptome data to refine the location confidence interval of this AF QTL on GGA5 and to characterize it functionally. To reduce the cost of microarray experiments, only 46 animals randomly selected for AF values from the 71 BC2 male offspring were studied. Using a 20 K chicken oligo array (Ark-genomics), 20461 gene expression measurements were obtained from the livers of these animals. One microarray was discarded from the 46 because of lower hybridization signals, confirming the good quality of the technical procedures (see Materials and Methods), and leaving a total of 45 birds for further study. Fifty-five percent of the 20461 genes (11213) were selected as expressed in the liver (see Materials and Methods), among which a human ortholog with a HUGO symbol for 4002 genes was determined. The raw and normalized microarray data were deposited in the Gene Expression Omnibus (GEO) public repository [25]. The accession number for the series is GSE12319 and the sample series can be retrieved with accession numbers GSM309564 to GSM309609.

**Figure 1:**
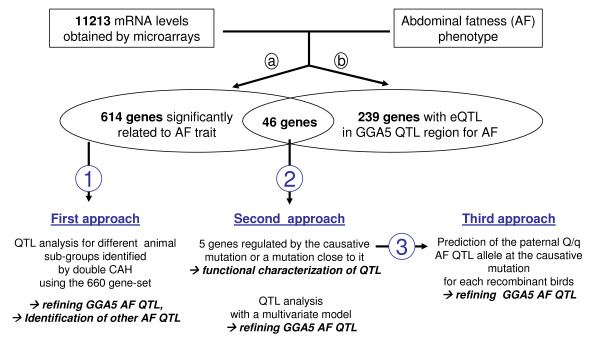
**Synthetic view of the different transcriptome approaches used to refine abdominal fatness QTL on GGA5**. **(a) **Correlation and differential expression analysis revealed 660 gene mRNA levels related to abdominal fat values (P < 0.05 at the gene level). **(b) **Expression QTL analysis performed on 11213 genes detected 285 genes for which mRNA levels were regulated by an eQTL which colocalized with the GGA5 AF QTL confidence interval (CI). Venn diagram shows that 46 gene mRNA levels were correlated with AF value and regulated by an eQTL colocalized with the AF QTL confidence interval. **(1) **First approach: QTL analysis was performed on different animal subgroups identified by double HCA carried out with the 45 animals and the 660 gene mRNA levels. **(2) **Second approach: we performed 46 new QTL analyses for residual abdominal fat values conditioned by each of the 46 gene mRNA levels. We thus identified 12 genes for which AF values conditioned by their mRNA level did not allow detection of residual AF QTL (p > 0.1), 5 of which were validated by qRT-PCR methodology. A multivariate analysis was then carried out combining a synthetic variable for the 5 gene mRNA levels and the AF trait to refine the QTL region of interest. **(3) **Third approach: We used the 5 gene mRNA levels to predict the Q/q allele at the causative mutation for each recombinant by discriminate analysis (DA) or logistic regression (LR). Supplementary marker genotyping localized in the AF QTL confidence interval made it possible to define the most probable AF confidence interval QTL. Aims for each approach are indicated in bold.

Preliminary linkage analysis on this 45 offspring subgroup revealed the expected AF QTL on the distal GGA5 with a significant effect of 1.03 phenotypic standard deviation and no QTL on GGA1, 3 and 7. Its location confidence interval (CI) extended from 156 cM to 187 cM, with the most probable location at 173 cM. This CI was in agreement with the 165-184 cM CI previously detected in a F2 design of 1300 birds [26]. The overall strategy to improve characterization of the distal GGA5 AF QTL is shown in Figure 1 and began with first selecting genes correlated with the trait of interest.

### Selection of 660 genes "correlated" with the AF trait

Six hundred sixty genes were found to be "associated" with the AF trait by analysis of their correlation with AF (P < 0.05 at the gene level) or their differential expression between the 10 fattest and 10 leanest birds out of the 45 (P < 0.05 at the gene level). Despite the absence of correction for multiple tests, principal component analysis (PCA) generated with the 660 transcript levels showed appropriate separation between fat and lean chickens on the two principal components, explaining 30% of the data variance (Additional file 1).

### Approach 1: Refining GGA5 AF QTL by dissection of the AF complex trait using the 660 gene-set transcriptome profiles

**Figure 2:**
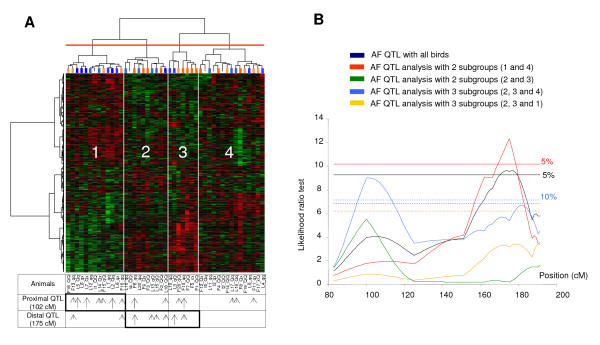
**Two-way Hierarchical Cluster Analysis (HCA) of the 45 animals and 660 gene-set (A) and AF QTL analyses (B)**. **(A) **HCA color matrix display obtained with the 660 genes (Y axis) and the 45 chickens (X axis). Dark/light blue bars indicate the 20 fattest chickens (dark bars correspond to the extreme fat chickens (F1 to F10), light bars the next (F11 to F20)); dark/light orange bars indicate the 20 leanest chickens (dark bars correspond to the extreme lean chickens (L1 to L10), light bars the next L11 to L20); colorless bars correspond to the 5 intermediate chickens (I). The two final letters of the animal labels, indicate the Q or q haplotype inherited from the sire, with a probability > 95% for the QTL at 102 cM (first letter) or the QTL at 175 cM (second letter); × indicates a probability < 95%. For the two QTL, animals with discrepant AF values and q/Q haplotype are indicated by arrows. Long arrows indicate the 10 most extreme animals with AF value in discordance with q/Q haplotype. Short arrows indicate the 10 lowest extreme animals (F11 to F20 and L11 to L20). **(B) **Interval mapping for the AF trait on chromosome 5, with the whole family (blue) and without one or two subgroups observed by HCA (other colors). The chromosome-wide significance thresholds at the 5% level (-) are displayed. The 10% level (- -) obtained for analysis without subgroups 1 (light blue), 4 (yellow) or 1 and 4 (green) are also displayed. The genetic distances (cM) and likelihood ratio test (LRT) are shown on the X-axis and Y-axis, respectively.

This approach aimed at "dissecting" the AF trait by separating the offspring into homogenous subgroups using the 660 transcript profiles. We performed two-way hierarchical cluster analysis (HCA) on the 45 offspring and the 660 genes related to AF. The double HCA presented in Figure 2A distinguished four bird groups. Initial observation of these subgroups showed that most of the 10 leanest birds were in subgroup 1 (6/10) whereas the 10 fattest birds were distributed randomly. *W*e then performed separate QTL analyses on the population, removing one or two groups from the four. As shown in Figure 2B, linkage analysis on AF with the GGA5 markers using groups 1 and 4 showed a slight increase in LRT (12.6) at 174 cM, which contributed to a small reduction in the location confidence interval for GGA5 AF QTL from 156 cM-187 cM (31 cM) to 158 cM-184 cM (26 cM). This QTL vanished by linkage analysis of subgroups 2 and 3. Interestingly, the linkage analysis with two subgroups 2 and 3 suggested an AF QTL at 102 cM on GGA5. We previously detected this QTL using a larger experimental design (1300 birds) [26]. The addition of subgroup 4 to subgroups 2 and 3 made this QTL significant (Figure 2B) with an effect on the AF trait of 1.19 phenotypic standard deviation. No AF QTL was detected for other subgroup combinations on GGA1, GGA3 or GGA7.

In order to improve the understanding of the characteristics of the subgroups obtained by HCA, we calculated (on the basis of the marker information only) the probability of each offspring receiving from its sire the Q or q haplotype for the two QTL at 102 cM and 175 cM; we considered only birds having a probability > 0.95 (43 and 35 birds for the two QTL at 102 cM and 175 cM, respectively). For the two AF QTL detected, the animal labels on Figure 2A show the distribution of birds for which the AF value was in disagreement with the paternal Q/q haplotype. A large proportion of these birds were included in subgroups whose suppression allowed an increase in power of QTL detection (subgroup 1 for the QTL at 102 cM and subgroups 2 and 3 for QTL at 175 cM). These results may explain the better power of linkage analysis using this "subgroup" strategy.

### Approach 2: refining distal GGA5 AF QTL by eQTL mapping

#### Selection of genes correlated with the AF trait and having an eQTL colocalizing with GGA5 AF QTL

Out of the previously selected 660 genes, we identified 46 genes (6.9%) that had an eQTL (p-value < 0.1) that colocalized with the location confidence interval of the GGA5 AF QTL (156-187 cM). Among the 11213 genes analyzed, 285 genes (2.3%) had an eQTL that mapped in the GGA5 AF QTL. Therefore, using the 660 genes correlated with the AF trait led to a 3-fold increase in genes (6.9% against 2.3%) having an eQTL colocalizing with the GGA5 AF QTL. The same analysis was performed on the other chromosomes (GGA1, GGA3 and GGA7), and no enrichment of genes with an eQTL in a particular region of these chromosomes was observed in this 660 gene-set, probably because of the absence of other AF QTL regions on these chromosomes. As previously observed with the AF QTL analysis, the design of 45 animals allowed us to detect significant eQTL regions with an effect on an expression trait of about 1 within-family residual standard deviation, with probably some false positives (no correction for multiple tests). Maximum likelihood ratio test (LRT) locations of eQTL for the 46 previously selected genes were evenly distributed over the 156-187 cM location confidence interval of the GGA5 AF QTL. Correlations between gene expressions were variable (from ~0 up to 0.7 and -0.64), suggesting different independent gene networks. These results strongly suggested that this 46 gene-set reflected at the mRNA level the impact of different linked mutations in the location confidence interval of the GGA5 AF QTL.

#### Selection of the genes regulated by the GGA5 AF QTL mutation or a mutation close to it

To select the closest eQTL mutations to the location of the GGA5 AF QTL mutation, we performed 46 linkage analyses on the residual AF value corrected for each transcript. Genes could be sorted by this step according to the degree of correlation between their transcript levels and AF trait and/or the high proximity of the maximum LRT location for their eQTL with one of the AF QTL. As a result, 12 genes out of the 46 genes effectively corrected the AF QTL (P > 0.1).

#### Validation by RT-PCR

Because of the high probability of false positives (no correction for multiple tests) we needed to validate these results using another method of mRNA quantification. Expression of the 12 genes was quantified by qRT-PCR. The previous results were confirmed for 5 genes. This 5 gene-set was considered to be the best gene-set giving new information about the position of the causative mutation underlying AF QTL and possibly about the impact at the mRNA level of this mutation for some of them. We therefore used these 5 genes to refine confidence interval of AF QTL location and also analyzed their functions to identify those likely to be regulated by the mutation itself.

#### Functional analysis of the 5 gene-set

According to the V3.2 annotation of the 20 K chicken oligochip (see Materials and Methods), 3 of the 5 genes of interest had a precise gene name: HGMCS1, TCF3 and SALL4. RIGG10516 and RIGG19646 genes both encode a hypothetical protein. A gene-by-gene bibliography indicated that TCF3 and SALL4 are both transcriptional factors involved in large molecular processes such as B-cell development and pluripotent stem cell generation, respectively. Neither seems to be directly involved in lipid metabolism. However, HGMCS1 (3-Hydroxy-3-MethylGlutaryl-CoA Synthase 1) is clearly involved in lipid metabolism, more precisely in cholesterol synthesis [27].

#### Refining GGA5 AF QTL by mapping of the 5 gene-set combined variable

**Figure 3:**
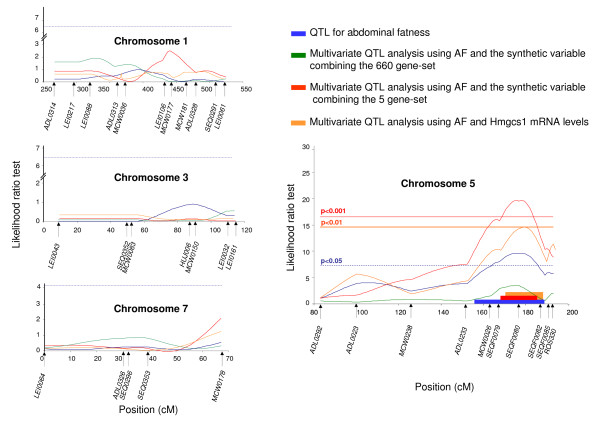
**Detection on chromosomes 1, 3, 5 and 7 of QTL for AF trait using a single trait or multi-trait model**. The multi-trait model concerned the AF trait combined with the HMGCS1 gene or the synthetic variable combining the 660 gene-set or the 5 gene-set. The chromosome-wide significance threshold at the 5% level for the AF QTL analysis (- - -) is indicated by a dashed blue horizontal line. On chromosome 5, the 1‰ level threshold (-) for the multivariate QTL analysis using AF and the synthetic variable combining the 5 gene-set is indicated by a red line. The genetic markers and genetic distances (cM) are shown on the X-axis. The likelihood ratio test (LRT) is shown on the Y-axis.

To take advantage of the correlation between the 5 expression traits and the AF trait and of their eQTL localized close to GGA5 AF QTL, we applied a multivariate model to refine GGA5 AF QTL. QTL detection with such a model can be more powerful and more precise than a single trait detection [28]. Because multivariate analysis is time consuming, we before generated a new synthetic variable (CV5) combining the 5 genes. Already used in the same context by Lan *et al*. [29], Principal Component Analysis (PCA) was appropriate to reduce the dimensions of the gene expression data. We then added together all 5 gene variables by weighting them with their PCA coordinates on the first axis. The first axis that explained 41.2% of the data variance effectively separated the 10 extreme fat and lean offspring, confirming that the GGA5 AF QTL had a substantial effect on the AF trait in this family (Additional File 2A). Note that HMGCS1 and RIGG10516 contributed most to this first axis (Additional File 2B). Indeed HMGCS1 and RIGG10516 expressions were the most highly correlated with the AF trait among the 46 gene-set (0.42 and -0.43, respectively). We then performed QTL detection using a multivariate model [28] considering the combined variable (CV5) and AF trait. As indicated in Figure 3, we then detected a QTL on GGA5 at 176 cM with high significance (P < 0.001). No QTL was detected on the other three chromosomes (GGA1, GGA3 and GGA7) (Figure 3). The maximum LRT was considerably increased for the multivariate analysis (19.9) compared to the univariate analysis with the AF trait only (10.2). These results led to a reduction of the location confidence interval of GGA5 AF QTL (from 156-187 cM (31 cM) to 166-184 cM (18 cM)) (Table 1), thus allowing a substantial reduction in numbers of positional candidate genes (from 100 to 46).

**Table 1:** Summary of reduction of GGA5 AF QTL using the 3 approaches

Confidence interval (CI) of GGA5 AF QTL according to the strategy used	cM^1^	Significance level^2^	CI (cM)^3^	CI(Mb)^4^	Gene number^5^	QTL effect/SD^6^
**Initial AF QTL CI**	**173**	*	**156-187 (31)**	**52-58 (6)**	**100**	**1.03**

First approach	175	*	158-184 (26)	52.2 - 57 (4.8)	74	1.56
Second approach	176	***	166-184 (18)	54 - 57 (3)	46	/
Third approach	/	/	166-173 (7)	54.16 - 55.1 (1.04)	12	/

**Overlapped CI taking into account all approaches**	**/**	**/**	**166-173 (7)**	**54.16-55.1 (1.04)**	**12**	/

^1 ^The most probable location for QTL in Kosambi cM. ^2 ^Chromosome-wide significance levels (* = P < 0.05; *** = P < 0.001). ^3 ^Location of 95% CI in cM according to drop off method. Total length (in cM) of CI is indicated in brackets. ^4 ^Location of 95% CI in Mb. Total distance (in Mb) for the CI extrapolated to the closest markers is indicated in brackets. ^5 ^Gene numbers present in the 95% CI according to NCBI database source. ^6 ^SD: within-family residual standard deviation.

The next step was to use these 5 genes to refine the location of this QTL with another approach (Figure 1).

### Approach 3: Refining distal GGA5 AF QTL by prediction of the paternal Q/q GGA5 AF QTL mutation, using the 5 gene-set for the recombinant birds

**Figure 4:**
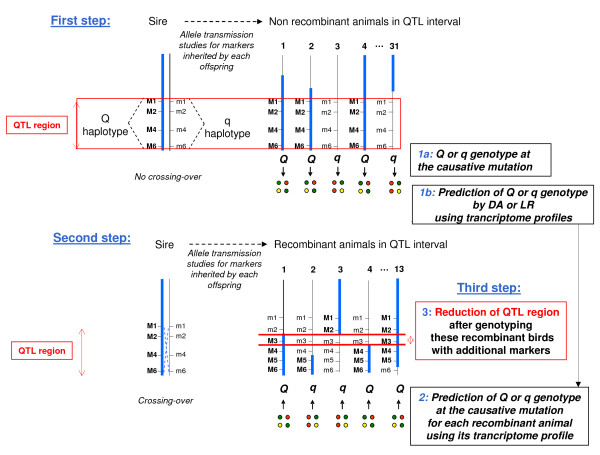
**Summary of the third strategy**. This approach was divided into two major steps. First, non-recombinant animals in the GGA5 AF QTL confidence interval were selected. Discriminant analysis (DA) or Logistic Regression (RL) were then carried out with the 5 genes to distinguish a specific transcriptome profile between the Q or q allele at the causative mutation. Second, previously established DA or RL were used to predict a most probable Q or q allele at the causative mutation for each recombinant animal in the GGA5 AF QTL confidence interval. New markers were then developed and genotyped (not shown on the figure) to define each recombination breakpoint. Comparison between recombinant offspring then enabled us to define the most valuable location of the causative mutation (line in red).

The aim of this approach was to predict the GGA5 AF QTL mutation (Q versus q) for the recombinant animals inherited from the sire, using their transcriptome profiles for the 5 gene-set considered to be the best signature of the Q/q AF QTL haplotype containing the Q/q mutation. The principle of this strategy is summarized in Figure 4. First, we determined the Q versus q haplotype (corresponding to the whole confidence interval of the GGA5 AF QTL) inherited from the sire for all offspring, using only the marker genotypes in the GGA5 AF QTL confidence interval as information. We thus determined with certitude such a haplotype for 32 offspring: 16 animals received the paternal QTL Q haplotype and 16 the q haplotype. For these offspring, the Q (versus q) haplotype contained with certitude the Q (versus q) GGA5 AF allele at the causative mutation. Second, using the transcriptome profiles for the 5 genes previously detected as the best possible signature of the location of Q versus q GGA5 AF QTL mutation, we looked for the best gene expression combination discriminating the 16 Q from the 16 q animals using two methods, *g.e*. discriminant analysis and logistic regression (see Methods). Third, we used the results of this analysis to predict the paternal Q versus q allele received by each recombinant animal, using their 5 gene-set transcriptome profiles. The two methods gave the same allele prediction with high probability (> 88%) for 8 recombinants out of the 13 (I3, L2, L19, F5, F9, F14, L13 and L6, presented in Additional File 3). To determine recombination breakpoints, we then developed three novel microsatellite markers between *SEQF0079 *and *SEQF0082 *at 166 and 187 cM, respectively, and genotyped the 5 most interesting recombinant animals among the 8 (Additional File 3). As indicated in Table 1, the Q or q allele prediction for these animals made it possible to refine the CI of the GGA5 AF QTL at 7 cM (166-173 cM). The physical locations of this interval were 54.16 to 55.2 Mb (1 Mb) on GGA5. This significant reduction was consistent with the reductions obtained by the other two approaches. Thus the number of best positional candidate genes was reduced from 100 to 12 genes, of which 3 have a precise gene name (AKT1, CDC14, NUDT14). No biological relationships with HMGCS1 were clearly identified.

## Discussion

The first approach consisted of "dissection" of the complex AF trait by grouping the offspring in accordance with their 660 gene transcriptome profiles. Schadt *et al*. [10] were the first to use this strategy and they improved the significance of a fatness QTL previously detected on a chromosome and even detected a new QTL on another. We found similar results in the present study. First, we found an increase in the LRT of GGA5 AF QTL by using subgroups 1 and 4. Second, despite the small size of our experimental design (45 animals), we detected using subgroups 2 and 3 an AF QTL at 102 cM on GGA5, as previously detected using an experimental design with 1300 birds [26]. These results show the power of the approach. In view of the polygenic influence on the complex traits, the variations in abdominal fatness are probably due to variations in several biological pathways, impacted by multiple mutations acting separately or in interaction. Transcriptome data offer the possibility of dissecting such a complex trait in more elementary phenotypes (gene expressions correlated with the trait) and therefore make it possible to separate the population into genetically homogenous subgroups using transcriptome profiling. Some combinations of subgroups possibly reflect the effects of a precise mutation whereas others reflect the signature of other mutations. In this study, we showed the over representation in some subgroups of birds for which the AF value was in disagreement with the paternal Q/q haplotype of the two GGA5 AF QTL, thus increasing the QTL detection power when they were removed. These results indicate that the QTL each significantly affect only a subset of the population analyzed. This heterogeneity observed between offspring clearly demonstrates the complexity underlying traits such as fatness. In summary, the 660 genes correlated with the AF allowed classification of offspring in a relevant way to dissect AF QTL on the GGA5 chromosome, despite the small number of animals analyzed.

To the best of our knowledge, no study using this approach has been published since the first publication in 2003 [10]. Our results obtained with a livestock design and those of Schadt *et al*. obtained with mice indicate that the identification of subgroups in a population on the basis of transcriptome profiles would be an effective way to improve the power of QTL detection by linkage analysis.

The second strategy aimed at improving characterization of GGA5 AF QTL by eQTL mapping. Such a strategy is widely used in the context of QTL detection of complex traits using transcriptome data [10,11,19,23,30-37]. The principle is to identify genes correlated with the complex trait that have an eQTL co-localizing with the QTL of interest. Most of these authors then focused on genes with a function related to the complex trait and having a cis-eQTL, allowing them to hypothesize that the mutation responsible for the complex trait is in the cis-eQTL gene [10,30,32,34-37]. In our study, no cis-eQTL was detected among the 46 genes correlated with the AF trait and having an eQTL in the GGA5 AF QTL region. Correlation pairs between these genes strongly suggested that these 46 trans-eQTL gene expressions were probably controlled by different mutations in the location confidence interval of the GGA5 AF QTL, as previously commented by Georges [6] and Schadt *et al*. [8]. Because the CI of the QTL was large (31 cM), we therefore selected 5 genes that effectively corrected the AF QTL (P > 0.1), and therefore likely to be controlled by the mutation sought or by a mutation close to it. Functional analysis of these 5 genes was still limited by the partial functional annotation of genes. However, we identified one gene related to lipid metabolism that could be affected by the mutation sought. This gene encodes HGMCS1, known to be involved in cholesterol metabolism. We have recently shown that its regulation in response to fasting is different in chickens compared to mammals [38]. Further experiments will be necessary to clarify its role in fatty acid metabolism and its regulation in chickens in order to target a potential regulatory gene in the distal GGA5 AF QTL.

Moreover, the whole 5 gene-set considered as a signature of the mutation underlying the QTL of interest or a mutation close to it may be useful to refine this QTL using a multivariate model that takes advantage of the correlation between these 5 expression traits and AF. Multivariate analysis combining the CV5 variable and the AF trait led to a significant increase in maximum LRT compared to the AF trait. This result supported the hypotheses of the existence of QTL affecting both AF and CV5 at the same position or the existence of different close mutations in linkage disequilibrium. We were unable to reach a conclusion with such a small number of animals analyzed. However, this result makes it possible to reduce the location confidence interval of GGA5 AF QTL from 156-187 cM (31 cM) to 166-184 cM (18 cM).

Finally, an original approach to refine a QTL region was proposed in this study. We used the same 5 gene-set to find the best gene expression combination discriminating the paternal Q from q haplotypes (corresponding to the whole confidence interval of the GGA5 AF QTL) and used it to predict the Q versus q mutation received by the recombinant animals. Genotyping these birds with additional markers drastically reduced the region to 166-173 cM (7 cM). Contrary to conventional approaches used to refine a QTL, such a strategy avoided generating new offspring to test the QTL genotype of the recombinant birds and saved on high levels of genotyping, thus gaining time and saving money. However, it is important to remember that it was based on the relevance of the gene-set considered as the signature of the QTL mutation or mutations close to it.

The gains in power and precision of QTL detection offered by approaches 1 and 2 were probably limited by the low density of markers and size of the experimental design used in this study (45 birds). Nevertheless, these approaches allowed substantial reduction of the GGA5 AF QTL region (20% up to 50%). Approach 3 was more effective (80% reduction), depending on the recombination breakpoints in the recombinant birds. This third approach allowed us to refine the GGA5 AF QTL from 156-187 cM (31 cM) to a most probable location confidence interval of 166-173 cM (7 cM) (Table 1). It can be seen that this reduction of GGA5 AF QTL region was consistent with the other more limited reductions obtained by the other two approaches. Unfortunately, a gene by gene bibliography analysis did not allow us to propose a good functional and positional gene candidate as regulator of HMGCS1 and the AF trait.

## Conclusion

Our results showed the value of using "Genetical Genomics (GG)" to characterize QTL responsible for complex trait variability in livestock. The originality of this study was to propose complementary approaches allowing a reduction of a QTL region and also providing functional information on it. The most common way to use GG in the QTL detection field is to identify an eQTL region colocalizing with a QTL of interest, making it possible to propose candidate genes possibly regulated by the QTL mutation. In the present study, we identified HGMCS1 that could be affected by the GGA5 AF QTL. In addition, as previously reported by Schadt *et al*. [10] using a different animal design, we showed that the identification of animal subgroups on the basis of their transcriptome profiles is an effective way to partially eclipse the polygenic effects which interfere in classical QTL analyses. Such an approach improves the precision of previously detected QTL and also localizes new ones. Finally, the original procedure, consisting of predicting QTL mutation allele for recombinant animals using a haplotype signature based on transcriptome profiles, may lead to drastic reduction of the QTL region. Detection of causative gene mutations underlying the GGA5 AF QTL will be further studied by increasing marker density in the QTL region and the number of animals analyzed, the number of recombinants being crucial in QTL detection analyses. Because of the availability in the future of high marker density combined with the drastic price reduction of microarrays, larger eQTL experimental designs are expected in livestock and this should accelerate identification of causative genes responsible for economic trait variability.

## Methods

### Animals and experimental procedures

All animals were bred at INRA, avian experimental unit UE1295, F-37380 Nouzilly, in accordance with European Union guidelines for animal care and under the authorization 6290 delivered to Nadine Sellier by the French Ministry of Agriculture. A three-generation design was generated by-intercrossing two experimental meat-type chicken lines that were divergently selected on fatness [39], referred to as fat (FL) and lean (LL) lines. First generation F1 birds were generated from 14 FL males mated with 24 LL females and 4 LL males mated with 8 FL females. A full genome scan in one F2 experimental design bird allowed us to identify several QTL for AF and to refine one of them located on chromosome 5, and 81 backcross (BC1) males were produced from an F1 male (known to be heterozygous for the GGA5 AF QTL) mated with 10 LL females. One of the BC1 animals, recombinant for the paternal chromosome in the QTL region, was mated with 8 LL females to generate 71 male BC2 animals obtained in four hatches. Progeny testing demonstrated that this male was heterozygous for the GGA5 AF QTL and was therefore chosen for transcriptome analysis. Forty-six animals were randomly chosen from the 71 birds to be analyzed by microarray procedures. BC2 chickens were fed ad libitum using a conventional starter diet (0-3 weeks: 12.8 MJ of metabolizable energy) and then a growing broiler diet (4-9 weeks: 13.0 MJ of metabolizable energy). Light/dark periods were 24 h light for the first 2 days, then 14 h light/10 h night up to slaughtering. At 4 weeks of age, blood samples were collected for DNA extraction and genotyping. At 9 weeks of age, the birds were fed ad libitum for a minimum of 4 hours after overnight fasting and then weighed and sacrificed by electrical stunning in the experimental processing plant. Following sacrifice, livers were collected, quickly frozen in liquid nitrogen and stored at -80°C until RNA extraction for transcriptome analyses. After evisceration, carcasses were stored overnight at 4°C before dissection and weighing of abdominal fat.

### DNA extraction and marker genotyping

Genomic DNA was extracted from blood samples (100 μl) from the 71 male BC2 according to the modified phenol/chloroform method [40]. DNA was quantified by the saran method [41] or optical density reading and diluted to 10 ng/μL. Genotyping for chromosomes 1, 3, 5 and 7 was performed for 11, 7, 10 and 5 markers, respectively (Additional Files 4 and 5). Fluorescence-labelled microsatellites were analyzed on an ABI 3100 DNA sequencer (Applied Biosystems, Foster City, CA, USA). Genotypes were interpreted using both the GenoMapper™ software (Applied Biosystems, Foster City, CA, USA) and the GEMMA database [42]. The Kosambi genetic distances in centi-Morgans (cM) were newly estimated with all animals using the "build" option in the CriMap linkage program [43]. Marker order was explored using the FLIPS command until the marker order that maximized the likelihood was obtained.

### RNA isolation

Total RNA was extracted with TRIzol^® ^reagent (Invitrogen, Cergy Pontoise, France) according to the manufacturer's instructions. Quality and concentration of extracted RNA were assessed using a 2100 Bioanalyzer (Agilent Technologies, Massy, France).

### Microarray procedures

#### Array slides

The 20 K chicken array printed in singlets was produced by ARK-Genomics (Roslin Institute - UK: http://www.ark-genomics.org), and the array design was published in the ArrayExpress [44] repository with Accession N° A-MEXP-820 ArrayExpress 2003 and in the Gene Expression Omnibus with the name GPL5480 [25]. The functional annotations used in the present study (version V3.2) are available on the web site: http://www.sigenae.org. They were obtained by a bioinformatics procedure developed by SIGENAE (INRA-France) [45].

#### mRNA labeling, hybridization and data acquisition

All these procedures were as previously described by Desert *et al*. [38]. Briefly, 5 μg of each mRNA sample were reverse-transcribed and Cy5 fluorescence-labeled. Each Cy5-labeled mRNA sample was hybridized to the microarray with the same Cy3-labeling reference probe according to the procedure of Transcriptome-Biochip Laboratory of Genopole "Toulouse Midi-Pyrénées" (France). The reference RNA pool was made up from equal amounts of RNA derived from all liver samples. The fluorescence ratio for each gene reflected the relative abundance of the mRNA of interest in each experimental sample compared with the same reference mRNA. The reference thus made it possible to take into account any eventual "spot × array" interaction.

Fluorescence signals were detected with a laser scanner (GenePix 4000A from Axon Instrument, CA) keeping a constant PMT gain for each channel. The images were then analyzed with GenepixPro 4.0 software (Axon instruments, Inc., Union City, CA). The raw files were stored in genepix files compatible with the LIMMA library of the R-project statistical and Bioconductor environment [46] which was used for the normalization and analysis of the data.

#### Data filtering

The first step was to select the genes considered as expressed in the liver (roughly 60% expected) showing a good contrast between spot and background intensities (SNR (Signal to Noise Ratio) defined as greater than 2). Among the 20461 genes on the microarray, we selected 11590 genes (57%) and eliminated one microarray for which the average SNR was equal to 1.5. We then removed 377 genes for which a minimum of 20% of spots among the 45 microarrays did not respect the 2 criteria: i) absence of the genepix flag (automatically performed by GenepixPro 4.0 [47]) and ii) symmetry of the spot. A total of 11213 genes were finally selected. Finally we targeted the isolated "bad" spots that did not conform to the two criteria in order to avoid taking them into account during the normalization procedure (2.8% of spots).

#### Data normalization

The procedure was previously described by Desert *et al*. [38]. The Cy5/Cy3 ratio used was expressed as the Log2 of the ratio of median pixel intensity of the red and green spots. Median Log2 ratio values were then normalized (ratio centered on zero) according to the hypothesis that the majority of gene expressions did not differ between two samples. The normalization was performed by a nonlinear regression method (Lowess fitness) [48] to take into account the intensity dependence of the fluorescence bias.

#### Data analyses

Raw data analyses were performed using a code written in R and softwares from the open-source Bioconductor Project [46]. The animal labels were defined as follows: F1 to F20 for the 20 fattest animals, L1 to L20 for the 20 leanest animals and I for the 5 intermediates. Analysis of variance between the 10 fattest (F1-F10) and 10 leanest chicken (L1-L10) groups for the AF trait value were performed with "aov" function. Two-way Hierarchical Cluster Analysis (HCA) was performed using the "hclust" function with "1-cor" as distance function and "ward" as aggregation criterion; the "heatmap" function was used to generate images. Principal Component Analysis (PCA) was performed with the "pca" function of the FactoMiner library. The predictions of Q versus q allele at the QTL mutation for the recombinants were estimated by discriminant analysis or a logistic regression model using the "lda" and "glm" functions of R, respectively.

### Real time quantitative RT-PCR (qRT-PCR) assay

Reverse transcription (RT) was carried out using the high-capacity cDNA archive kit (Applied Biosystems, Foster City, CA) according to the manufacturer's protocol. Briefly, 200 μL of each reaction mixture containing 20 μL of 10× RT buffer, 8 μL of 25× dNTPs, 20 μL of 10× random primers, 10 μL of MultiScribe Reverse Transcriptase (50 U/μL), and total RNA (10 μg) was incubated for 10 min at 25°C followed by 2 h at 37°C. A 1/10 or 1/20 dilution (depending on the gene) of each RT reaction was further used for real time quantitative PCR (qPCR). cDNA samples were mixed with 20 μl ABsolute SYBR Green Mix (Abgene, UK) and 300, 450 or 600 nM (according to the gene) of specific reverse and forward primers. Reaction mixtures were incubated in an iCycler iQ Multicolour Real-Time PCR Detector (Bio-Rad, Marne la Coquette, France) programed to perform one cycle (95°C for 15 min) and 40 cycles (95°C for 15 s and 59°C for 45 s). A melting curve program was then performed for each gene to check the presence of a single product with a specific melting temperature. For each sample and each gene, PCR runs were performed in duplicate. For each gene, serial PCR reactions constructed with 2-fold serial dilutions from a pool of the cDNA samples were systematically added on each microplate for the calibration curve and determination of the amplification rate (R) of the Taq polymerase. For all genes, the amplification rates were within the range 99% to 100% and could be considered as equal to 1. Thus, for the same sample, the gene expression level could be normalized relative to the *B-actin *expression level.

### QTL and eQTL mapping

Before QTL analyses, the AF trait values of the sire family (71 birds) were adjusted for hatch and dam effects by two-way variance analysis, including body weight at slaughter as a covariate (SAS GLM procedure). For the eQTL analyses, no adjustment of the gene variables was performed for hatch and dam effects because of the small size of the population studied (45 birds). QTLMAP software based on an interval mapping method described by Elsen *et al*. [49], was used to detect QTL (or eQTL) affecting the AF trait (or a gene expression phenotype). Gene expression values were obtained by microarray or qRT-PCR procedure. The statistical variable for testing the presence of one QTL (or eQTL) *versus *no QTL (or no eQTL) at one location was an approximate likelihood ratio test (LRT) [50]. Significance thresholds were empirically determined for AF QTL and transcript level eQTL from 2000 simulations. The widely used "one LOD drop-off method" was applied to obtain 95% confidence intervals of the QTL location [51]. QTLMAP software was also used to perform multivariate QTL analysis. As only two traits (CV5 and AF) was studied, we were able to apply a multivariate model with a multinormal penetrance distribution, which is the most powerful and accurate method, even though it is time consuming [28].

## Abbreviations

AF: Abdominal Fatness; BC: Backcross; CI: Confidence Interval; CV: Combined Variable; DA: Discriminant Analysis; GEO: Gene Expression Omnibus; GGA: *Gallus Gallus*, GO: Gene Ontology; GG: Genetic Genomics; HCA: Hierarchical Cluster Analysis; KEGG: Kyoto Encyclopedia of Genes and Genomes; HMGCS1: 3-Hydroxy-3-MethylGlutaryl-CoA Synthase 1; HUGO: Human Genome Organization; LR: Logistic Regression; LRT: Likelihood Ratio Test; PCA: Principal Component Analysis; qRT-PCR: quantitative Reverse Transcription-Polymerase Chain Reaction; eQTL: Expression Quantitative Trait Locus.

## Authors' contributions

Transcriptome data acquisition was supervised by SL. GLM recorded, analyzed and interpreted the transcriptome data, supervised by SL. FP and SoL performed genotyping. BA and SL carried out the preliminary QTL mapping analyses on backcross families. GLM carried out the QTL/eQTL mapping analyses supervised by PLR and SL. GLM and GG performed the discriminant analysis and logistic regression for predicting the recombinant haplotypes. GLM and SL drafted the manuscript. All authors read and approved the final manuscript.

## Supplementary Material

Additional File 1 **Principal Component Analysis (PCA) for the 45 animals with the 660 gene-set**. The gene variables for the PCA were scaled to give them the same importance. X-axis and Y-axis represent the first and second principal components that explained 21.7% and 10% of animal dispersion, respectively. **(A) **Individual factor map. The 20 extreme fat and lean animals (F1-F10 and L1-L10) are indicated in red and blue, respectively. The next 20 fat and lean birds and intermediate animals are indicated in black. **B: **Gene factor map. Click here for file

Additional File 2 **Principal Component Analysis (PCA) for the 45 animals with the 5 gene-set**. The gene variables for the PCA were scaled to give them the same importance. X-axis and Y-axis represent the first and second principal components that explained 41.2% and 23% of animal dispersion, respectively. **(A) **Individual factor map. The 20 extreme fat and lean animals (F1-F10 and L1-L10) are indicated in red and blue, respectively. The next 20 fat and lean birds and intermediate animals are indicated in black. **B: **Gene factor map. Click here for file

Additional File 3 **Prediction of the paternal Q or q allele at the causative mutation for recombinant animals and estimation of the most valuable location for AF QTL**. On the basis of genetic marker information only, we determined for each recombinant animal the Q (orange box) or q haplotype (green box) inherited from its sire covering the 36 cM confidence interval of the distal GGA5 AF QTL. White box represents undetermined Q or q haplotype region. Q or q allele at the causative mutation for each recombinant animal was then determined by the discriminant analysis (DA) or logistic regression model (LR) (see Methods) using its 5 gene-set transcriptome profiles. All the recombinants (lines in gray) for which the probability of the prediction by the two methods was not greater than 88% were not considered. This prediction for each recombinant helped us to isolate the most probable region for causative mutation location. Black dotted boxes represent regions for which the localization of the causative mutation was excluded. Taking recombinants together, the most probable causative mutation location is indicated by a blue square. New markers next genotyped (*SEQALL0402*, *SEQF0081 *and *SEQALL0540*) are indicated in blue italic letters to define the recombination breakpoints. Comparison between F5, F9, F14, L13 and L6 recombinant offsprings then helped us to define the most valuable location of the causative mutation (new blue square). ^1 ^Microsatellite marker names located on the distal part of chromosome 5; in italics the markers added to determine recombination points. ^2 ^Location for each marker in centiMorgan (cM) or Megabase (Mb).^3 ^Q or q haplotype inherited from the sire; "0" indicates undetermined paternal allele. ^4 ^AF values for each recombinant animal are shown as a rough guide. ^5 ^and ^6 ^Allele predicted at the causative mutation (Q or q) by discriminant analysis (DA) or logistic regression model (LR) using the 5 gene-set, respectively. "/": undetermined allele. Click here for file

Additional File 4 **International genetic markers used**. Markers were chosen from international available markers (Groenen et al, 2000, *Genome Res*, 10:137-147), or developed for this program (Abasht et al 2006, *Genet Sel Evol*, **38**(3):297-311; see additional file 5). Click here for file

Additional File 5 **New microsatellite markers developed from the chicken genome assembly**. (galGal3, http://genome.ucsc.edu/cgi-bin/hgGateway). Click here for file
