# Supplementary material for: Using transcriptome profiling to characterize QTL regions on chicken chromosome 5
Source: BMC Genomics. 2009 Dec 2;10:575. doi: 10.1186/1471-2164-10-575 (PMC2792231; doi:10.1186/1471-2164-10-575)
Supplement: Additional File 1 — Principal Component Analysis (PCA) for the 45 animals with the 660 gene-set. The gene variables for the PCA were scaled to give them the same importance. X-axis and Y-axis represent the first and second principal components that explained 21.7% and 10% of animal dispersion, respectively. (A) Individual factor map. The 20 extreme fat and lean animals (F1-F10 and L1-L10) are indicated in red and blue, respectively. The next 20 fat and lean birds and intermediate animals are indicated in black. B: Gene factor map. [file 1471-2164-10-575-S1.PPT]

## Slide 1
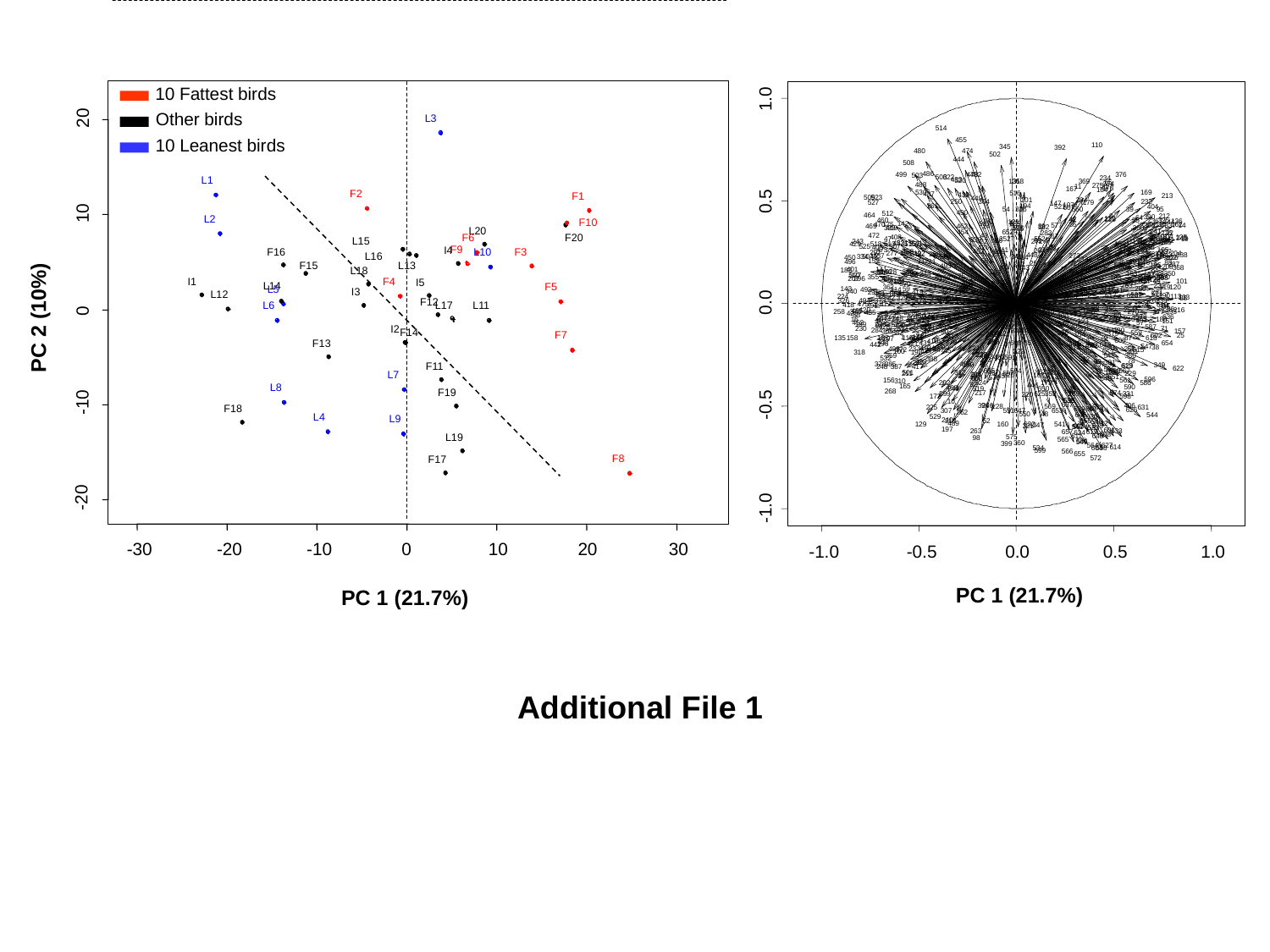

10 Fattest birds
1.0
Other birds
20
L3
514
480
474
502
508
486
499
478
482
503
506
520
488
516
487
509
523
521
490
512
464
460
517
470
469
462
459
485
472
518
513
515
507
473
458
457
504
500
484
496
493
463
510
481
492
483
495
511
494
476
475
477
491
465
522
471
505
479
461
498
466
501
467
497
468
519
489
10 Leanest birds
455
392
444
433
439
449
394
404
390
398
454
408
422
432
413
429
405
426
411
402
448
440
450
401
410
395
427
438
419
443
451
415
436
434
418
391
420
397
393
435
423
456
445
407
424
425
403
452
428
414
412
441
416
437
430
431
421
442
400
447
453
409
417
446
396
406
399
110
65
107
95
64
121
114
89
62
104
61
111
117
77
63
106
58
69
105
67
73
116
93
102
112
83
68
86
97
96
66
75
109
72
122
101
120
119
59
74
113
82
88
84
81
80
60
91
118
103
70
123
57
76
94
71
90
115
79
87
85
100
78
99
108
92
98
345
376
358
369
374
325
383
367
337
333
380
364
339
348
330
353
342
338
334
341
332
346
343
368
328
386
344
377
355
363
370
362
340
354
329
326
373
336
389
375
371
366
335
327
365
382
356
379
372
361
359
350
388
381
385
357
378
349
387
384
351
324
352
331
347
360
322
275
317
304
261
316
283
267
311
272
276
279
264
292
277
273
257
282
287
303
301
294
309
293
260
285
296
278
271
286
302
288
280
314
259
291
281
295
265
274
258
270
306
323
308
290
266
305
315
284
289
313
297
300
319
318
320
312
298
310
268
299
269
307
262
321
263
L1
234
213
201
250
232
194
212
219
247
241
226
245
252
242
243
206
237
192
204
238
191
195
208
235
207
209
203
231
236
190
224
205
239
233
255
254
216
200
230
253
193
251
196
214
249
210
256
222
198
227
228
223
215
199
248
211
229
221
202
244
217
220
246
225
240
218
197
131
178
167
164
169
179
147
132
136
144
142
175
162
182
176
125
185
159
130
134
126
152
141
183
146
163
161
138
168
124
143
150
139
145
137
133
181
174
177
170
148
188
155
151
184
189
149
171
180
157
186
154
140
135
187
158
153
127
166
156
173
165
172
128
129
160
11
F2
530
527
560
585
577
528
552
531
525
535
543
581
539
540
588
533
563
571
537
582
556
526
570
568
578
554
524
587
559
580
536
547
589
553
548
532
562
555
567
558
545
576
561
586
574
538
569
573
551
550
544
529
546
541
579
542
557
583
575
565
584
549
564
534
566
572
F1
0.5
10
607
652
592
649
634
641
617
628
638
610
636
609
648
644
642
602
618
606
603
654
643
616
615
593
591
613
629
622
594
635
621
597
620
623
651
600
646
596
590
650
619
625
598
595
637
631
639
626
647
653
645
630
656
632
605
601
633
612
624
608
611
640
627
614
604
599
655
54
39
8
L2
42
F10
55
35
14
L20
22
F6
F20
43
31
18
L15
47
26
19
20
37
F9
I4
3
F16
F3
L10
34
56
L16
33
41
15
13
9
21
F15
L13
17
6
4
L18
24
50
32
27
I1
F4
I5
49
L14
F5
28
L5
I3
44
30
L12
7
0.0
F12
L17
L6
L11
PC 2 (10%)
0
5
46
36
2
53
16
40
29
I2
F14
F7
25
1
F13
659
38
23
45
F11
L7
51
L8
F19
-10
-0.5
10
F18
660
48
L4
L9
52
12
657
L19
658
F8
F17
-20
-1.0
-30
-20
-10
0
10
20
30
-1.0
-0.5
0.0
0.5
1.0
PC 1 (21.7%)
PC 1 (21.7%)
Additional File 1
