# Supplementary material for: Using transcriptome profiling to characterize QTL regions on chicken chromosome 5
Source: BMC Genomics. 2009 Dec 2;10:575. doi: 10.1186/1471-2164-10-575 (PMC2792231; doi:10.1186/1471-2164-10-575)
Supplement: Additional File 2 — Principal Component Analysis (PCA) for the 45 animals with the 5 gene-set. The gene variables for the PCA were scaled to give them the same importance. X-axis and Y-axis represent the first and second principal components that explained 41.2% and 23% of animal dispersion, respectively. (A) Individual factor map. The 20 extreme fat and lean animals (F1-F10 and L1-L10) are indicated in red and blue, respectively. The next 20 fat and lean birds and intermediate animals are indicated in black. B: Gene factor map. [file 1471-2164-10-575-S2.PPT]

## Slide 1
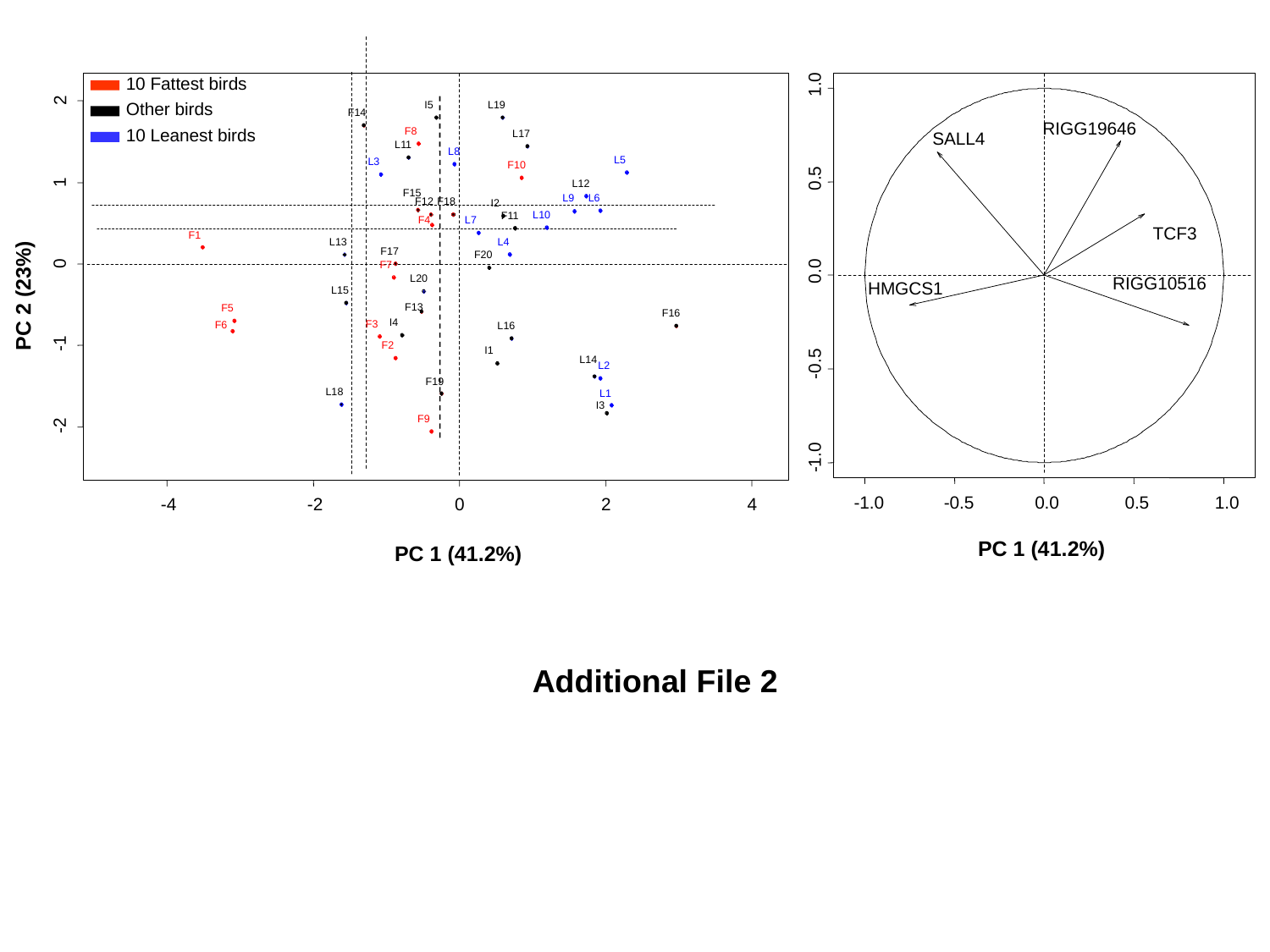

10 Fattest birds
1.0
2
Other birds
I5
L19
F14
RIGG19646
10 Leanest birds
SALL4
F8
L17
L11
L8
L5
L3
F10
0.5
1
L12
F15
L6
L9
F12
F18
I2
L10
F11
F4
L7
TCF3
F1
PC 2 (23%)
L13
L4
F17
F20
0
F7
0.0
RIGG10516
HMGCS1
L20
L15
F13
F5
F16
I4
F3
F6
L16
-1
F2
I1
L14
-0.5
L2
F19
L18
L1
I3
F9
-2
-1.0
-1.0
-0.5
0.0
0.5
1.0
-4
-2
0
2
4
PC 1 (41.2%)
PC 1 (41.2%)
Additional File 2
