# Supplementary material for: Using transcriptome profiling to characterize QTL regions on chicken chromosome 5
Source: BMC Genomics. 2009 Dec 2;10:575. doi: 10.1186/1471-2164-10-575 (PMC2792231; doi:10.1186/1471-2164-10-575)
Supplement: Additional File 4 — International genetic markers used. Markers were chosen from international available markers (Groenen et al, 2000, Genome Res, 10:137-147), or developed for this program (Abasht et al 2006, Genet Sel Evol, 38(3):297-311; see additional file 5). [file 1471-2164-10-575-S4.PDF]

Marker	Chromosome	Location (cM)
ADL314	1	281
LEI217	1	301
LEI088	1	317
ADL313	1	364
MCW036	1	386
LEI106	1	430
MCW177	1	440
MCW181	1	457
ADL328	1	475
SEQALL0291	1	502
LEI061	1	518
LEI043	3	9
SEQALL0352	3	50
MCW083	3	51
HUJ006	3	89
MCW150	3	90
LEI032	3	110
LEI161	3	113
ADL292	5	83
ADL023	5	100
MCW238	5	126
ADL233	5	151
MCW026	5	162
SEQF0079	5	166
SEQALL0402	5	173
SEQF0080	5	175
SEQF0081	5	176
SEQALL0540	5	182
SEQF0082	5	187
SEQF0085	5	192
ROS0330	5	198
LEI064	7	0
ADL326	7	30
SEQALL0296	7	33
SEQALL0353	7	39
MCW178	7	69

Additional file 4
