# Supplementary material for: Using transcriptome profiling to characterize QTL regions on chicken chromosome 5
Source: BMC Genomics. 2009 Dec 2;10:575. doi: 10.1186/1471-2164-10-575 (PMC2792231; doi:10.1186/1471-2164-10-575)
Supplement: Additional File 5 — New microsatellite markers developed from the chicken genome assembly. (galGal3, http://genome.ucsc.edu/cgi-bin/hgGateway). [file 1471-2164-10-575-S5.PDF]

Marker	Chr.	Location	Upper primer	Lower primer
SEQALL0291	1	181632608+181632887	GCACTTTATTGGCATCCACTC	GTCTTTGTGATGGCCTGAAC
SEQALL0352	3	13735645+13735815	TGTCTCCTTTTGGGCTTTTC	GAATTTGGAAGGAAAACATTTG
SEQALL0402	5	55205562+55205766	TAAGTGCAGGCAACAAACTG	AACAAGGTTCCTCCTGAAATG
SEQF0080	5	55425473+55425650	ACCTGTTAGCCATAAATACTG	AAATACAACAAAAGCTGTCAAG
SEQALL0540	5	56895719+56895904	TCAACTTTGGTCCTTTCTGC	AGTCTTTGTCCCTTCGTTCC
SEQALL0296	7	6174261+6174381	GCGTTTCCCACTCTCCTTCC	TTTTCCTCTCGTGGCATTTG
SEQALL0353	7	10861413+10861660	CATGATTCTGGCCTGTTTTC	ACAACAAAAGCCCCCACAG

Additional file 5
